# Characterization of dermal skin innervation in fibromyalgia syndrome

**DOI:** 10.1371/journal.pone.0227674

**Published:** 2020-01-13

**Authors:** Dimitar Evdokimov, Philine Dinkel, Johanna Frank, Claudia Sommer, Nurcan Üçeyler

**Affiliations:** Department of Neurology, University of Würzburg, Würzburg, Germany; Weill Cornell Medicine-Qatar, QATAR

## Abstract

**Introduction:**

We characterized dermal innervation in patients with fibromyalgia syndrome (FMS) as potential contribution to small fiber pathology.

**Methods:**

Skin biopsies of the calf were collected (86 FMS patients, 35 healthy controls). Skin was immunoreacted with antibodies against protein gene product 9.5, calcitonine gene-related peptide, substance P, CD31, and neurofilament 200 for small fiber subtypes. We assessed two skin sections per patient; on each skin section, two dermal areas (150 x 700 μm each) were investigated for dermal nerve fiber length (DNFL).

**Results:**

In FMS patients we found reduced DNFL of fibers with vessel contact compared to healthy controls (p<0.05). There were no differences for the other nerve fiber subtypes.

**Discussion:**

We found less dermal nerve fibers in contact with blood vessels in FMS patients than in controls. The pathophysiological relevance of this finding is unclear, but we suggest the possibility of a relationship with impaired thermal tolerance commonly reported by FMS patients.

## Introduction

A reduction of the intraepidermal nerve fiber density (IENFD) is one of the commonly reported findings [1–4], while dermal innervation apparently does not differ from healthy controls [4].

Most researchers use antibodies to the pan-axonal marker protein gene product 9.5 (PGP9.5) to determine skin innervation [5,6]. However, dermal and epidermal nerve fibers comprise diverse subpopulations, which can be differentiated by specific surface protein markers, e.g. peptidergic and non-peptidergic [7], and which differ in function. Data on cutaneous nerve fiber subpopulations are scarce. Furthermore, it is an open question, how a reduction in the number of peripheral nociceptors may be associated with more pain. While we found no difference in dermal innervation using antibodies to PGP9.5 [4], the composition of dermal fibers, some of which give rise to epidermal endings, might be different. While no such data are available for the dermis, in the epidermis, a decrease of non-peptidergic nerve fibers and an increase of peptidergic nerve fibers was demonstrated in rats after painful peripheral nerve injury [8,9]. In another study, chronic constriction injury of the sciatic nerve resulted in epidermal denervation of the rat skin at the time of maximum pain behavior, including fibers immunoreactive to calcitonin gene-related peptide [10]. Hence, we investigated dermal nerve fiber composition of patients with fibromyalgia compared to healthy controls as potential contributor to small fiber pathology in FMS.

## Methods

### Subject recruitment and clinical assessment

Between September 2014 and March 2017, we recruited 86 women with FMS (median age 51, range 23–74 years), who were also part of a large clinical study [11]. Inclusion criteria were age ≥18 years and diagnosis of FMS according to current criteria [12–14]. Exclusion criteria were polyneuropathy, pain of other origin and indistinguishable from FMS, diabetes mellitus, untreated thyroid dysfunction, renal insufficiency, rheumatic disorders as diagnosed by a rheumatologist, neurotoxic medication, and alcohol or substance abuse. All patients were individually seen, interviewed, and thoroughly examined by a neurologist. Pain distribution was determined by interview. Nerve conduction studies of the sural and tibial nerves were performed following standard procedures [15] to exclude polyneuropathy. Laboratory tests (including full blood count, electrolytes, kidney and liver function tests, thyroid stimulating hormone, vitamin B12, HbA1c, oral glucose tolerance test) were performed to exclude alternative causes of small fiber damage.

Additionally, we recruited 35 healthy women as control subjects (median age 49, range 22–66 years) for skin biopsies (see below). Controls were mostly acquaintances or friends of the patients. Inclusion criteria for healthy controls were: no neurological diseases, no pain or symptoms of neuropathy (i.e. sensory disturbance, persis), no metabolic or psychiatric diseases, normal neurological examination, normal neurographies of the sural and tibial nerve. All study participants gave written informed consent before study inclusion. Our study was approved by the Würzburg Medical Faculty Ethics Committee.

### Skin punch biopsy

Six-mm skin punch biopsies were obtained from the lateral lower calf (10 cm above ankle) as described earlier [16]. Skin samples were incubated in 4% paraformaldehyde (PFA; pH 7.4; 2 h) before processing for nerve fiber quantification on 40-μm cryosections.

### Immunohistochemistry

To visualize intraepidermal and dermal nerve fibers, we immunoreacted skin sections with antibodies against PGP9.5 (516–3344; 1:1000, Zytomed, Berlin, Germany) visualized by a Cy3-coupled secondary antibody (109-165-043; 1:100, Dianova, Hamburg, Germany). To further subclassify nerve fibers, we applied antibodies against calcitonin gene-related peptide (CGRP, ab81887, 1:100, abcam, Cambridge, United Kingdom) and substance P (SP, ab14184, 1:500, abcam, Cambridge, United Kingdom) as markers for peptidergic fibers, neurofilament 200 (NF200, F-1005, 1:200, Aves, Tigard, Oregon, USA) as a marker for myelinated fibers, and CD31 (550389, 1:500, BD Pharmingen, San Diego, California, USA) as a marker for endothelial cells. A secondary antibody coupled to Alexa Fluor 488 (DDXCH 03A488-100; 1:100, Dendritics, Lyon, France) was used. Except for PGP9.5, none of the tested antibodies delivered a reliable immunolabelling of epidermal nerve fibers in extensive pilot experiments. Sections were viewed using a fluorescence microscope (Axiophot 2, Zeiss, Oberkochen, Germany) equipped with an Axiocam mrm camera (Zeiss, Oberkochen, Germany) and SPOT software, Windows version 4.5 (Diagnostic Instruments, Inc, Sterming Heights, Michigan, USA).

### Quantification of IENFD

To determine the IENFD, three skin sections per biopsy site were assessed by an investigator unaware of subject allocation [17]. Data were compared with our laboratory normative values based on 106 healthy women (median age: 50 years, range: 20–84). The cut-off value for the IENFD at the lower leg was set at <6 fibers/mm.

### Quantification of dermal innervation

For quantification of dermal nerve fiber length (DNFL), we used a published method with slight modifications [18]. We assessed two skin sections per subject and on each section two dermal areas measuring 150 μm in depth x 700 μm in length (S1 Fig), i.e. four dermal areas per subject. As shown previously [18], DNFL/mm epidermis and DNFL/mm^2^ both correspond very well and can be used equally for quantification of dermal innervation. Each dermal nerve fiber in the corresponding area was tracked manually at 40x magnification and the total DNFL was divided by the length of the epidermis of the respective measured area (i.e. 700 μm). Hereby, we measured the whole length of the nerve which showed close proximity to a blood vessel at some points. For double immunoreactions, overlay with PGP9.5 was checked in every single nerve fiber for accuracy. In case of CD31, which is abundantly expressed on the surface of endothelial cells of blood vessels [19], we assessed nerve fibers contacting blood vessels, i.e. a nerve fiber was categorized as contacting a blood vessel, if a contact was seen on CD31 and PGP9.5 double stains. The length of dermal blood vessels was determined as described for DNFL above. Dermal nerve fiber quantification was performed in a blinded manner as to subjects group allocation.

### Statistical analysis

The Kolmogorov-Smirnov test showed normal distribution of dermal innervation data. Means were compared using the unpaired two-tailed Student`s t-test; data are presented as mean +/- standard deviation. Non-normally distributed data (IENFD, fibers with vessel contact and SP positive fibers) were analyzed using the Mann-Whitney U test. For correlation analysis between DNFL and IENFD; the Spearman correlation coefficient test was calculated. For comparison between categorical data the Chi^2^ test was used. Quantitative data are illustrated as box plots giving the median (black horizontal line in the box), and the 25^th^ and 75^th^ quartile (whiskers of the box). P-values <0.05 were assumed significant.

## Results

### Clinical data

Neurological examination, nerve conduction studies, and routine blood tests were normal in all patients. None of our patients reported symptoms or had clinical signs of polyneuropathy or radiculopathy. FMS patients and controls did not differ in age (p>0.05). For an overview of clinical data see Table 1.

**Table 1:** Clinical data of patient cohort.

	Fibromyalgia syndrome (n = 86)
Age [years]	51 (23–74)
BMI [kg/m^2^]	23 (16–42)
**FMS criteria fulfilled:**	
ACR 1990	78/86 (91%)
ACR 2010	86/86 (100%)
German S3 guideline	86/86 (100%)
Time since diagnosis [years]	4 (<0.5–35)
**Pain distribution:**	
Whole body	56 (65.1%)
Proximal distribution	26 (30.2%)
Distal distribution	2 (2.3%)
Not sure	2 (2.3%)

Median values are given with range in brackets. Abbreviations: BMI: body mass index, ACR: American College of Rheumatology.

### Skin innervation

**Fig 1:**
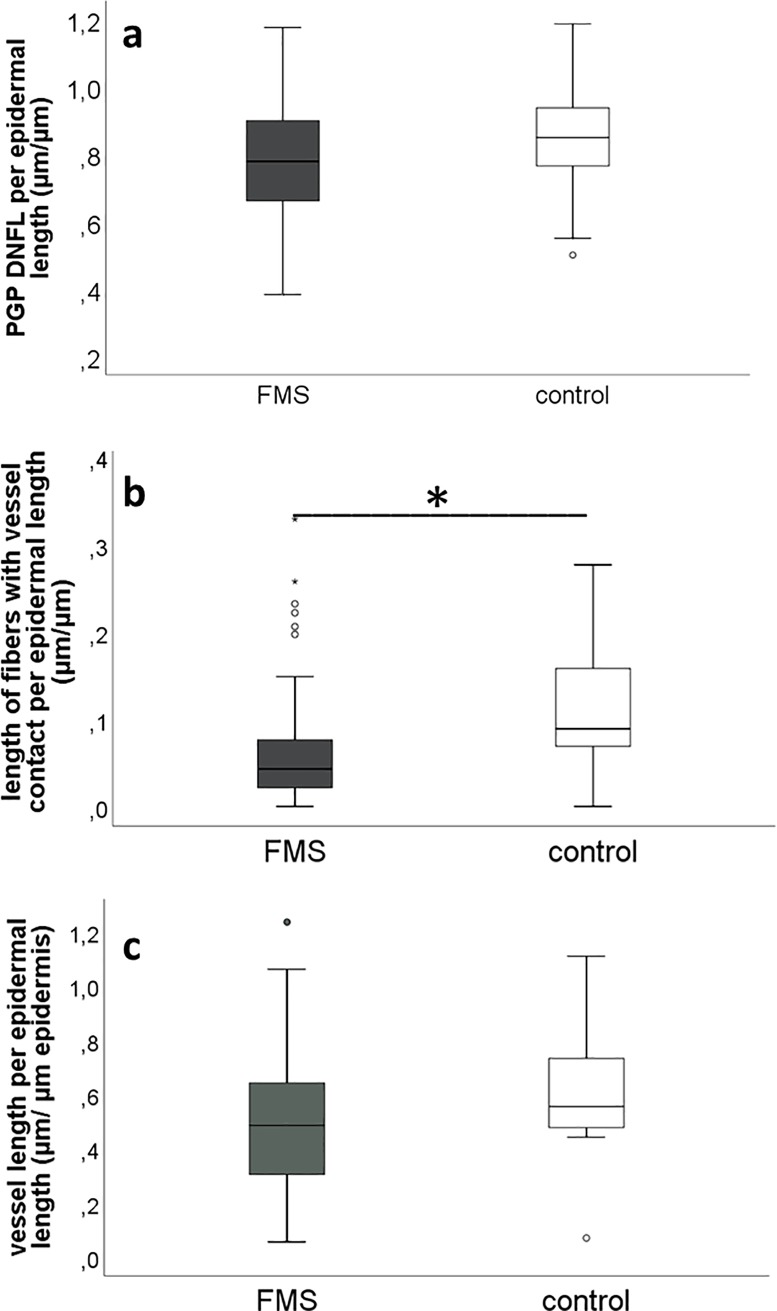
Quantification of dermal nerve fiber length (DNFL) and dermal nerve fibers with vessel contact. a: The DNFL did not differ between FMS patients and healthy controls. b: The length of dermal nerve fibers with vessel contact was reduced in FMS patients compared to healthy controls (p<0.05). c: The length of dermal blood vessels did not differ between FMS patients and controls. Abbreviations: DNFL: dermal nerve fiber length; FMS: fibromyalgia syndrome.

**Fig 2:**
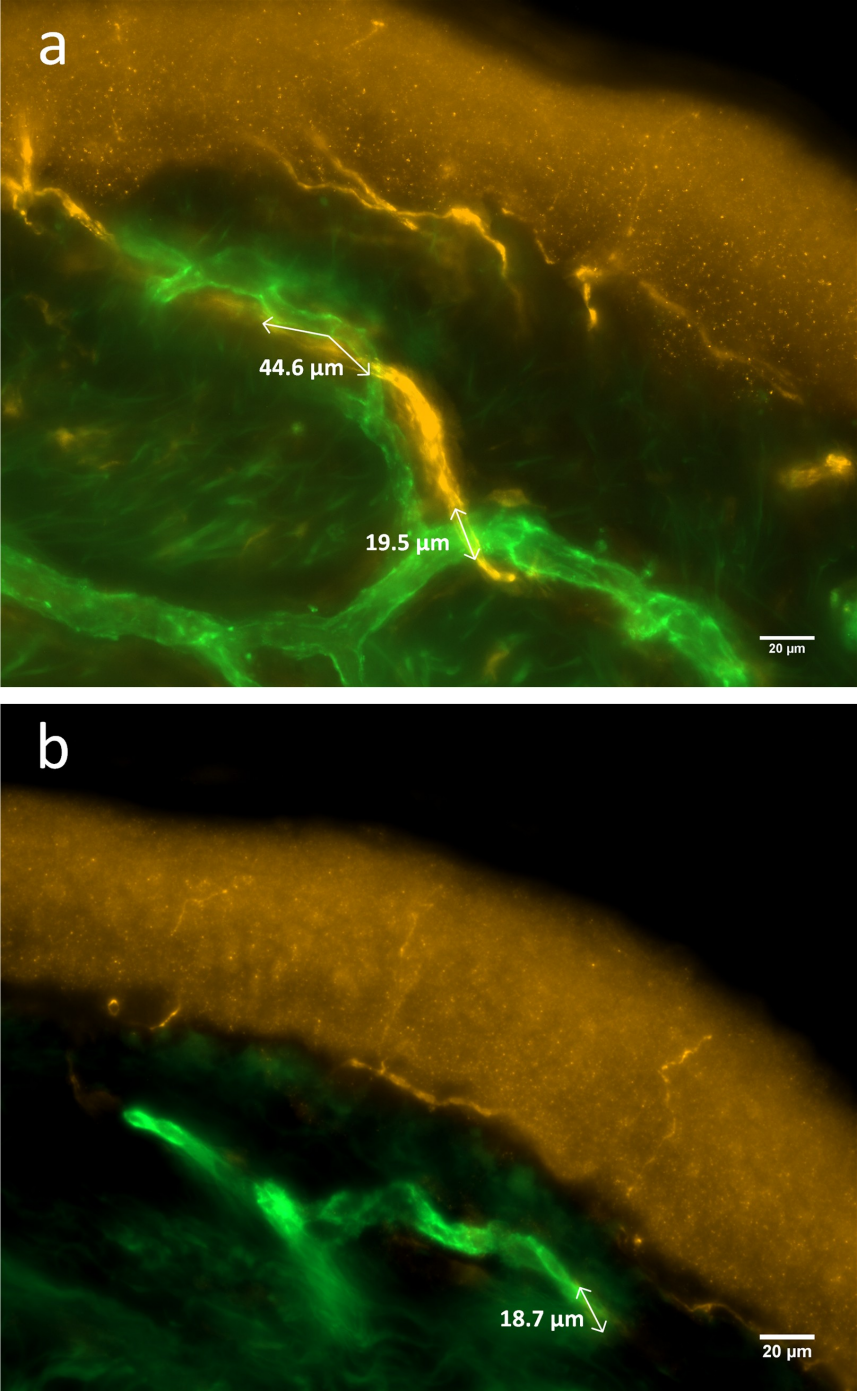
Dermal nerve fibers in contact to blood vessels in an FMS patient and a healthy control. Photomicrographs illustrate dermal nerve fibers in relation to blood vessels in a healthy control subject (a) and reduced numbers of nerve fibers in contact with dermal vessels in a patient with FMS (b). Skin sections are immunoreacted with antibodies against PGP9.5 (yellow signal) for cutaneous nerve fibers and CD31 (green signal) for dermal blood vessel endothelial cells. Arrows indicate nerve fibers innervating blood vessels. White arrows indicate sections with dermal nerve fibers coming in close vicinity to blood vessels. Scale bar: 20 μm. Abbreviations: FMS: fibromyalgia syndrome, PGP9.5: protein gene product-9.5.

IENFD was reduced in 38/86 (44%) patients while only in 7/35 (20%) healthy controls (Chi^2^ p<0.001). The overall DNFL immunoreacted with PGP9.5 did not differ between patients with FMS (median 0.78 μm/μm epidermis, range 0.38–1.18) and healthy controls (median 0.85 μm/μm epidermis; range 0.5–1.19; n.s.; Fig 1A), and also not between FMS patients with normal and reduced distal IENFD. However, total length of dermal nerve fibers with contact to blood vessels was lower in skin of patients with FMS (median 0.043 μm/μm epidermis, range 0–0.33) compared to healthy controls (median 0.089 μm/μm epidermis, range 0–0.28, p<0.05; Fig 1B). Since median vessel length did not differ between groups (FMS: 0.48 μm/μm epidermis, range 0.06–1.23; controls: 0.55 μm/μm epidermis, range 0.07–1.11; Fig 1C), findings for vessel innervation were independent (Fig 2).

**Fig 3:**
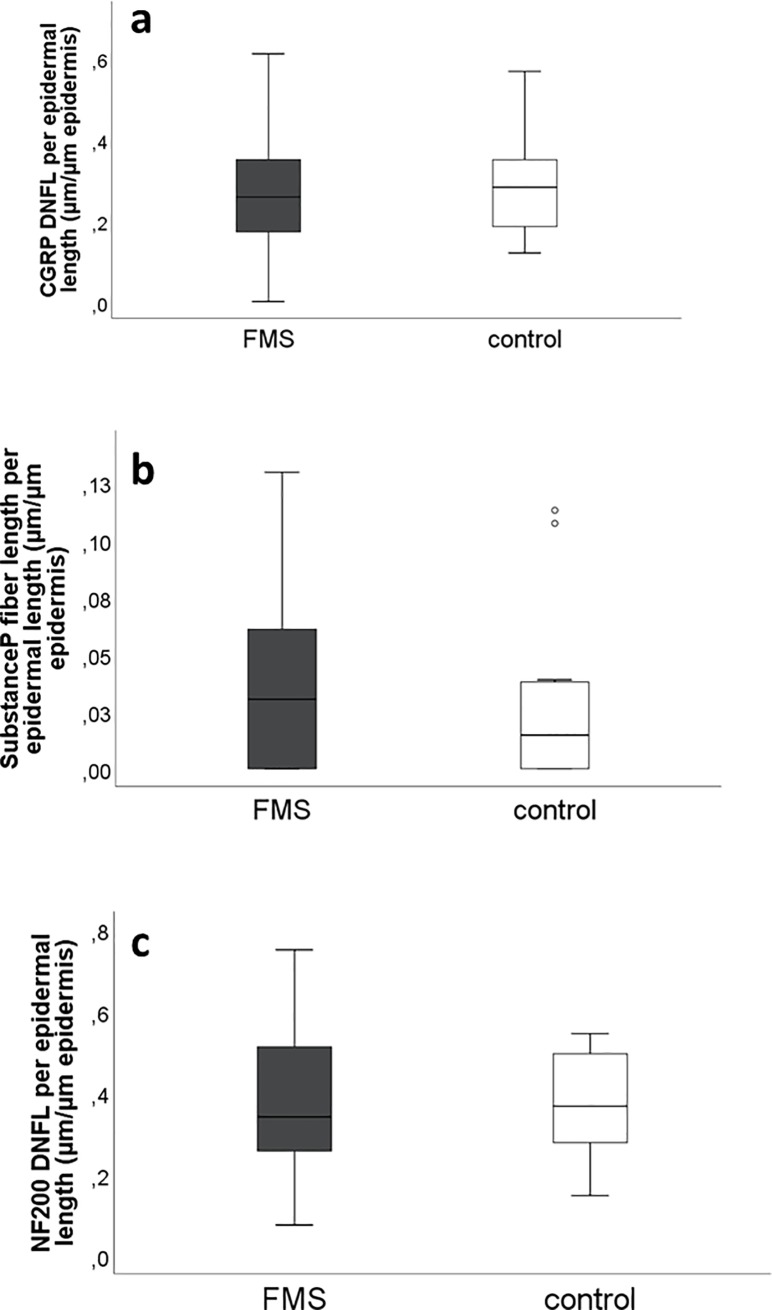
CGRP-, SP- and NF200-positive fibers. a: The length of dermal CGRP-positive nerve fibers did not differ between FMS patients and controls. b: The length of dermal SP-positive nerve fibers did not differ between FMS patients and controls. c: The length of dermal NF200-positive nerve fibers did not differ between FMS patients and controls. Abbreviations: CGRP: calcitonine gene-related peptide; FMS: fibromyalgia syndrome; NF200: neurofilament 200; SP: substance P.

Median length of peptidergic nerve fibers immunoreactive to antibodies against CGRP (FMS: 0.26 μm/μm epidermis, range 0–0.61; controls: 0.28 μm/μm epidermis; range 0.12–0.57; Fig 3A) and SP (FMS: 0.03 μm/μm epidermis, range 0–0.13; controls: 0.014 μm/μm epidermis, range 0–0.11; Fig 3B) did not differ between groups. The median length of NF200 positive myelinated nerve fibers was similar between FMS patients (0.34 μm/μm epidermis, range 0.075–0.75) and controls (0.37 μm/μm epidermis, range 0.15–0.55; n.s.; Fig 3C).

### Subgroup analysis

When investigating skin samples of FMS patients with normal and reduced IENFD at the calf, we found no difference in DNFL. Quantification of CGRP, SP, and NF200 immunoreaction also did not show intergroup differences. Also, we did not find correlations between clinical and histological data (data not shown).

## Discussion

We analyzed the epidermal and dermal innervation of FMS patients compared to healthy controls to assess if a potential shift in the composition of nerve fibers innervating the skin might be associated with pain in FMS. We found a reduced length of dermal nerve fibers in contact with blood vessels in the FMS patient group compared to controls, while dermal nerve fiber subtypes did not show intergroup differences.

So far, no data are available characterizing epidermal small fiber subpopulations in human skin due to lacking immunoreactivity of intraepidermal nerve fibers to antibodies directed against antigens other than PGP9.5. Two studies reported few peptidergic nerve fibers in the epidermis of healthy controls [20,21]. The majority of studies showed peptidergic nerve fibers almost exclusively in the dermis [22,23]. As previously reported [23], peptidergic CGRP positive nerve fibers are typically located in the subepidermal plexus and only occasionally enter the epidermis, whereas fibers positive for SP are sparse and rarely penetrate the epidermis.

In contrast to the epidermal fibers, dermal nerve fibers can be visualized more easily allowing the distinction between different fiber subpopulations. In patients with small fiber neuropathies, a reduction of DNFL was found as determined by immunoreaction of skin punch biopsy samples with PGP9.5 [18,24]. In these studies, DNFL also correlated with the IENFD.

In one study, the innervation of arteriole-venule shunts of hypothenar skin was assessed in FMS patients and healthy controls revealing that FMS patients had excessive peptidergic innervation [25]. In contrast, our study shows a reduction of vessel innervation in skin obtained from the lower leg. This discrepancy might be due to the biopsy site in terms of location (hand versus lower leg) and skin consistency (glabrous versus hairy skin). Also, the fact that we assessed all dermal vessels regardless of subtypes in contrast to arteriole-venule shunts in Albrecht et al. may have influenced the results.

We did not detect differences in nerve fiber length of other analyzed nerve fiber subtypes (PGP9.5, CGRP, SP, NF200), neither in the patient group as a whole, nor in the patient subgroup with reduced IENFD. This may indicate that alterations of skin innervation in FMS are more pronounced in the superficial epidermal layer than in the deeper dermal layer.

We failed investigating non-peptidergic nerve fibers lacking a sufficiently specific antibody against respective antigens and hence cannot rule out the possibility of intergroup differences. We found no evidence for a shift in the composition of nerve fiber subpopulations in our patient group compared to controls, but provide hints for reduced dermal blood vessel innervation. Further, it is still possible that the ion channel pattern of nociceptors is altered in FMS patients compared to controls, however, we could not test this hypothesis, since none of the ion channel antibodies we used displayed specific binding to skin nerve fibers. Further, we can only speculate on potential functional correlates of our data, since we did not perform respective tests such as the investigation of the axon reflex flare. However, our data are in line with previous studies giving hints for an altered peripheral circulation in FMS patients [26,27]. Our laboratory tests were limited to the main causes of small fiber impairment. We investigated biomaterial from only one biopsy site at the calf and did not include a second site from the thigh due to the laborsome individual and manual histological assessment. We cannot speculate on a potential influence of the body mass index (BMI) on our results, since we did not document the BMI from the controls. Also, the size of the control cohort for vessel innervation was n = 15, as some of the stains could not be assessed due to technical reasons. Further, it remains unclear if the nerve fibers observed in close vicinity to dermal blood vessel do innervate these vessels. Hence, data validation in a larger cohort and using advanced microscopy techniques is warranted. We speculate that the reduced number of dermal nerve fibers contacting blood vessels observed here may lead to an impaired vessel autoregulation, resulting in poor tolerance for hot and cold temperatures commonly reported by FMS patients [28]. Our data add to the growing evidence of a contribution of the peripheral nervous system to and a structural basis of FMS pathophysiology. Further research comparing FMS patients with and without reduced dermal vessel innervation is warranted to clarify the functional impact of our findings.

## Supporting information

S1 Fig Photomicrograph illustrating the assessment of dermal nerve fiber length. The analyzed dermal area starting at the dermal-epidermal border measured 150 μm in depth and 700 μm in length. Nerve fibers located in the marked four dermal areas were manually tracked.
(TIF)
