# Supplementary figures and images for: Characterization of dermal skin innervation in fibromyalgia syndrome
Source: PLoS One. 2020 Jan 13;15(1):e0227674. doi: 10.1371/journal.pone.0227674 (PMC6957156; doi:10.1371/journal.pone.0227674)

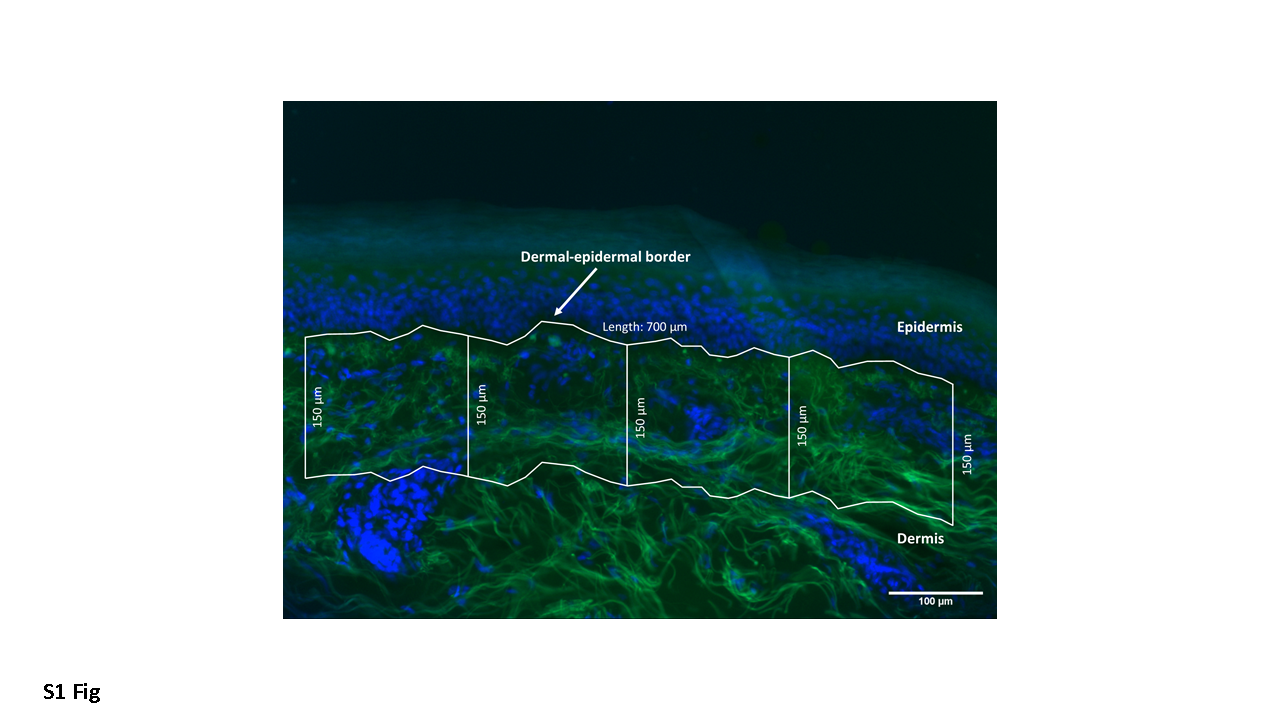

Supplement: S1 Fig — The analyzed dermal area starting at the dermal-epidermal border measured 150 μm in depth and 700 μm in length. Nerve fibers located in the marked four dermal areas were manually tracked. (TIF) [file pone.0227674.s001.tif]
